# 1126. Effect of Remdesivir on Recovery, Quality of Life, and Long-COVID Symptoms One Year after Hospitalization for COVID-19 Infection: A Randomized Controlled SOLIDARITY Finland Trial

**DOI:** 10.1093/ofid/ofac492.965

**Published:** 2022-12-15

**Authors:** Olli P O Nevalainen, Saana Horstia, Sanna Laakkonen, Jarno Rutanen, Jussi Mustonen, Ilkka Kalliala, Hanna Ansakorpi, Hanna-Riikka Kreivi, Pauliina Kuutti, Juuso Paajanen, Seppo Parkkila, Erja-Leena Paukkeri, Markus Perola, Negar Pourjamal, Andreas Renner, Tuomas Rosberg, Taija Rutanen, Joni Savolainen, Jari K Haukka, Gordon H Guyatt, Kari A Tikkinen

**Affiliations:** Faculty of Medicine University of Helsinki, Finland. Unit of Health Sciences, Faculty of Social Sciences, University of Tampere, Finland. Hatanpää Health Center, City of Tampere, Finland. Pirkanmaa Hospital District, PSHP, Tampere, Finland, Tampere, Pirkanmaa, Finland; University of Helsinki, Helsinki, Uusimaa, Finland; Faculty of Medicine, University of Helsinki, Finland, Helsinki, Uusimaa, Finland; Unit of Health Sciences, Faculty of Social Sciences, University of Tampere, Finland, Tampere, Pirkanmaa, Finland; Occupational Health Helsinki, Finland, Helsinki, Uusimaa, Finland; Department of Obstetrics and Gynecology, University of Helsinki and Helsinki University Hospital, Finland, Helsinki, Uusimaa, Finland; Department of Neurology, University of Oulu, Finland, Oulu, Pohjois-Pohjanmaa, Finland; Department of Respiratory Medicine, Heart and Lung Center, Helsinki University Hospital, Finland, Helsinki, Uusimaa, Finland; University of Helsinki, Helsinki, Uusimaa, Finland; Department of Respiratory Medicine, Heart and Lung Center, Helsinki University Hospital, Finland, Helsinki, Uusimaa, Finland; Faculty of Medicine and Health Technology, University of Tampere, Finland, Tampere, Pirkanmaa, Finland; Department of Internal Medicine, University of Tampere, Finland, Tampere, Pirkanmaa, Finland; National Institute for Health and Welfare (THL), Finland, Helsinki, Uusimaa, Finland; Faculty of Medicine, University of Helsinki, Finland, Helsinki, Uusimaa, Finland; Department of Respiratory Medicine, Heart and Lung Center, Helsinki University Hospital, Finland, Helsinki, Uusimaa, Finland; Department of Respiratory Medicine, Kanta-Hame Central Hospital, Hameenlinna, Finland, Hameenlinna, Kanta-Hame, Finland; Patient Partner, Helsinki, Uusimaa, Finland; Patient Partner, Helsinki, Uusimaa, Finland; Faculty of Medicine, University of Helsinki, Finland, Helsinki, Uusimaa, Finland; Department of Clinical Epidemiology and Biostatistics and Department of Medicine, McMaster University, Hamilton, ON, Canada, Hamilton, Ontario, Canada; Faculty of Medicine, University of Helsinki, Finland. Department of Urology, University of Helsinki and Helsinki University Hospital, Finland. Department of Surgery, South Karelian Central Hospital, Lappeenranta, Finland, Helsinki, Uusimaa, Finland

## Abstract

**Background:**

Coronavirus disease 2019 (COVID-19) patients frequently suffer from long-term sequelae, often called “long COVID” or “post COVID-19 condition”. Remdesivir, given in early disease, decreases the risk of hospitalization and potentially mortality. No randomized trials have thus far published long-term follow-up data on any COVID-19 drug treatment. We investigated the effects of remdesivir on a range of patient-important outcomes at one year.

**Methods:**

Between July 2020 and January 2021, an open-label randomized multicenter trial in Finland recruited 208 adult patients from 11 Finnish hospitals. Patients were randomly assigned (1:1 ratio) to standard of care (SoC)with remdesivir (median duration of remdesivir treatment 5 days) or SoC alone. Primary outcomes were self-reported recovery, exertional dyspnea, fatigue, and quality of life at one year. Secondary outcomes were overall mortality and several potential long-COVID symptoms.

**Results:**

At one year, 5 (4.4%) of 114 patients in the remdesivir and 5 (5.3%) of 94 in the SoC group had died (RR 0.82, 95% CI 0.25-2.76; absolute difference: -0.9%, 95% CI -7.9-5.3); 181 (92% of survivors) completed the follow-up. Self-reported recovery (fully or largely) occurred in 85% in remdesivir and in 86% in SoC (RR 0.94, 0.47-1.90; absolute difference: -0.9%, 95% CI -11%-10%). Exertional dyspnea occurred in 5% in remdesivir and 8% in SoC (OR 0.61, 95% CI 0.20-1.85; absolute difference -3.3%, 95% CI -12%-4.4%). We found no convincing difference between remdesivir and SoC groups in quality of life or symptom outcomes (p > 0.05 for all). Of the 21 potential long-COVID symptoms, patients often reported moderate or major bother from fatigue (26%), joint pain (22%), persistent respiratory mucus (21%), and problems with memory (19%) and attention/concentration (18%) (Figure).

Bother from potential long-COVID symptoms at one year from COVID-19 hospitalization between the standard of care and standard of care plus remdesivir groups.

**Figure:**
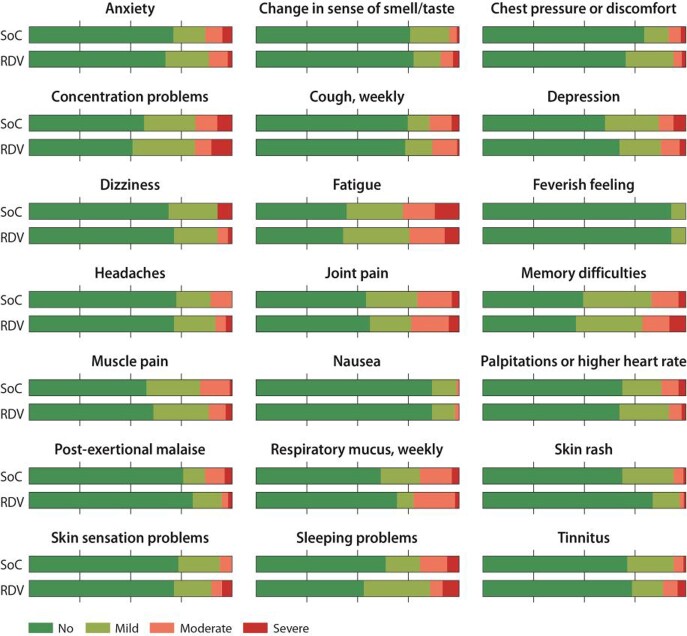

**Conclusion:**

After a one-year follow-up of hospitalized patients (with a very high participation rate), approximately one in four reported substantial bother from fatigue, and one in six reported that they had not recovered well from COVID-19. We found no convincing evidence of a remdesivir effect, but confidence intervals were wide and included possible substantial benefit and substantial harm.

**Disclosures:**

**Hanna-Riikka Kreivi, MD, PhD**, Pfizer: Advisor/Consultant|Roche: Advisor/Consultant **Tuomas Rosberg, MD, PhD**, AstraZeneca: Honoraria|Boehringer-Ingelheim: Honoraria|GSK: Honoraria.

